# The bidirectional relationship between severity of obsessive-compulsive symptoms and lifestyle factors in patients with obsessive-compulsive disorder: a contemporaneous and prospective analysis

**DOI:** 10.3389/fpsyt.2025.1552691

**Published:** 2025-07-21

**Authors:** Johanna A. M. du Mortier, Anton J. L. M. van Balkom, Jos W. R. Twisk, Patricia van Oppen, Henny A. D. Visser

**Affiliations:** ^1^ Department of Research, Mental Health Care Institute, GGz Centraal, Amersfoort, Netherlands; ^2^ Department of Psychiatry, Amsterdam University Medical Center (UMC), location Vrije Universiteit Amsterdam, Amsterdam, Netherlands; ^3^ Mental Health Program, Amsterdam Public Health, Amsterdam, Netherlands; ^4^ Department of Epidemiology and Biostatistics, Amsterdam University Medical Center (UMC), location Vrije Universiteit Amsterdam, Amsterdam, Netherlands; ^5^ Department of Psychiatry, Amsterdam University Medical Center, University of Amsterdam, Amsterdam, Netherlands

**Keywords:** obsessive-compulsive disorder, OCD, physical activity, life style, BMI, illicit drug, smoking

## Abstract

**Objective:**

In psychiatric disorders, lifestyle factors are known to influence both the development and course of the illness. However, little is known about the longitudinal relationship between lifestyle factors and OCD symptom severity, including the potential bidirectional, prospective association between them. This study examines both the contemporaneous and two-year prospective relationships between lifestyle factors and OCD symptom severity, as well as the reverse relationship—namely, the influence of OCD severity on lifestyle factors.

**Methods:**

Longitudinal data spanning six consecutive years were obtained from the Netherlands Obsessive Compulsive Disorder Association study (NOCDA). We examined the lifestyle factors: smoking, alcohol and illicit drug use, physical activity, and body mass index (BMI). Mixed models and Generalized Estimating Equations were employed to analyze the contemporaneous and bidirectional prospective relationships between these lifestyle factors and OCD severity.

**Results:**

Drug and alcohol use, BMI, and physical activity did not exhibit a significant contemporaneous relationship with OCD severity. Smoking was significantly associated with more severe OCD symptoms: however depression influenced this relationship. Using alcohol was significantly associated with lower OCD severity. Among females, alcohol use was significantly associated with lower OCD severity two years later. Moderate and high levels of physical activity were significantly associated with lower OCD symptom severity two years later, whereas other lifestyle factors did not significantly predict future OCD symptom severity. OCD symptom severity did not predict any lifestyle factor, except among females, where higher OCD severity was associated with lower drug use two years later.

**Conclusion:**

Previous studies on other psychiatric disorders have found that unhealthy lifestyle factors are associated with more severe psychiatric symptoms. It appears that OCD might differ in these aspects. In the present study, drug and alcohol use, higher BMI, and a composite score of unhealthy lifestyle factors were not associated with more severe OCD symptoms and did not predict greater symptom severity two years later. However, consistent with findings in other psychiatric disorders, higher levels of physical activity were significantly associated with lower OCD symptom severity two years later. Further research is needed to determine whether increasing physical activity could result in less severe OCD symptoms.

## 1 Introduction

In medical literature, a healthy lifestyle is typically defined as one that minimizes the risk of diseases such as cardiovascular disorders and cancer. The World Health Organization (WHO) recommends regular physical activity, no smoking, no alcohol use and maintaining a healthy Body Mass Index (BMI) to prevent cancer and cardiovascular diseases (1, 2). In the context of psychiatric disorders, physical activity, diet, and smoking are also considered important lifestyle factors which could influence the development and course of mental disorders (3). Given the tendency for unhealthy lifestyle factors to co-occur, several studies have focused on addressing multiple lifestyle factors simultaneously. For instance, a study conducted on a community sample in Madrid examined the presence of tobacco use, high-risk alcohol consumption, leisure-time inactivity, and unbalanced eating habits, finding that nearly 20% of the population met criteria for three or four unhealthy factors (4). Reflecting this co-occurrence, recent intervention studies aimed to improve multiple lifestyle factors simultaneously (5–7). Two meta-analyses found that interventions focusing on multiple lifestyle factors reduced the severity of anxiety (7) and depressive symptoms (5, 6) in the short term (one to three months) but not during longer follow-up (5–7).

Despite this knowledge, information about the lifestyle of individuals with OCD remains limited. While the prevalence of smoking appears to be lower in patients with OCD compared to the general population (8), OCD has been associated with a higher incidence of alcohol dependence (9). Conversely, OCD symptoms are frequently reported in individuals with alcohol dependence (10). The relationship between diet, BMI and OCD appears to be complex. A study including 85 adults with OCD showed that OCD severity had little effect on nutrient intake and dietary quality (11). Another study on OCD and BMI, involving 316 individuals with OCD, found that OCD was not associated with a lower or higher mean BMI compared to individuals with a depressive disorder, an anxiety disorder, or both, as well as non-clinical controls. However, a subset of individuals with OCD without depression was significantly associated with a lower BMI compared to individuals with OCD and depression (12).

Research exploring the impact of lifestyle on the course of OCD is similarly limited. In a randomized controlled trial (N=56), the effects of adding aerobic exercises to standard treatment were compared to adding health education. That study found that the Yale-Brown Obsessive Compulsive Scale (Y-BOCS) score significantly decreased post-treatment in both groups, but the decrease did not differ significantly between them (13). Another randomized controlled trial (N=125), in which participants were randomly assigned to four different groups (waitlist, CBT, CBT with exercise, and exercise only), found no significant differences in reduction of OCD severity between CBT with or without exercise. However, the total frequency of exercise predicted OCD symptom reduction in both the CBT with exercise and exercise only groups (14). Regarding alcohol use and the course of OCD, a study with 281 patients with OCD treated in intensive residential treatment, found that treatment responders showed lower past-year alcohol use scores than non-responders (15). To the best of our knowledge, there are no other studies on the course of OCD in relation to lifestyle factors and interventions addressing multiple lifestyle factors in individuals with OCD.

OCD has a high comorbidity with mood disorders. In a large study involving 2,073 individuals with OCD, it was found that 41% had received a lifetime diagnosis of major depressive disorder (16). Furthermore, there appears to be a relationship between OCD severity and the severity of depressive symptoms. In a study of 324 individuals with OCD, it was observed that more severe OCD predicted more severe depressive symptoms the following year (17). Depressive disorders have a significant influence on lifestyle factors and vice versa. Depression is associated with lower physical activity (18) and a higher body mass (19), and some lifestyle factors are linked to an increased risk of depression, such as smoking (20), alcohol use disorder (21), and cannabis use (22). Conversely, physical activity protects against the development of depression (23), and exercise can be used as a treatment for depression (24).

Given the scarcity of studies on lifestyle factors in individuals with OCD, this study aims to fill this gap by first examining the contemporaneous relationship between physical activity, smoking, alcohol and illicit drug use, BMI, the total number of unhealthy lifestyle factors, and the severity of OCD symptoms. We did not have information about the dietary habits of the individuals in our study; therefore, we examined BMI as a proxy for diet. Although the use of illicit drugs is not typically considered a lifestyle factor, we included it because its use is associated with a negative impact on mental functioning (22, 25, 26). Second, this study investigates the prospective bidirectional relationship between these lifestyle factors and the severity of OCD symptoms two years later. Because unhealthy lifestyle factors tend to co-occur, we also studied the contemporaneous and prospective bidirectional relationships between OCD and a composite score of unhealthy lifestyle factors. To our knowledge, this is the first study to assess multiple lifestyle factors—as well as a composite lifestyle score—while exploring both the contemporaneous relationship between lifestyle factors and OCD severity, and the prospective bidirectional relationship between these variables in a relatively large sample over a substantial time span.

Based on existing literature on lifestyle and OCD, as well as other mental disorders, we hypothesize that more severe obsessive-compulsive symptoms are associated with lower levels of physical activity, more smoking, higher alcohol and illicit drug use, and a higher BMI. We also expect that more severe OCD is associated with a greater number of unhealthy lifestyle factors in one person. Additionally, we anticipate that lower physical activity, smoking, alcohol and illicit drug use, and higher BMI will predict more severe OCD symptoms two years later, with an expected dose-response effect—indicating that having a lifestyle with multiple unhealthy factors will be associated with higher OCD symptom severity. Furthermore, we hypothesize that OCD symptom severity is associated with more unhealthy lifestyle factors two years later.

Due to the associations between OCD and depression, and between depression and lifestyle factors, we anticipated that depression could potentially amplify the relationship between OCD severity and low physical activity, smoking, alcohol use, and illicit drug use. Furthermore, while individuals with OCD without comorbid depression seem to have a lower BMI than those with OCD and depression (12), the presence of comorbid depressive disorders may act as a moderator, potentially altering the relationship between BMI and OCD severity. Therefore, we examined the relationships described above both with and without adjusting for comorbid depression, as well as with and without considering an interaction between depression and lifestyle factors.

This study is clinically relevant because, despite the availability of evidence-based treatments, OCD often follows a chronic course and is associated with significant burden and reduced well-being (27–32). If lifestyle factors influence OCD severity, incorporating lifestyle interventions could potentially enhance treatment outcomes. Conversely, if OCD negatively impacts lifestyle behaviors, it is important to identify this influence so that preventive or supportive interventions can be integrated into treatment to mitigate these effects.

## 2 Methods

The reporting of this study adheres to the Strengthening the Reporting of Observational Studies in Epidemiology (STROBE) statement (www.strobe-statement.org).

### 2.1 Procedure and quality aspects

The data for this study originated from the Netherlands Obsessive Compulsive Disorder Association (NOCDA) study, a longitudinal cohort investigation aimed at exploring the naturalistic long-term course of OCD in patients referred to seven mental health care centers. A comprehensive description of the NOCDA study design and the baseline characteristics of the study sample is available elsewhere (33). Ethical approval for the NOCDA study was obtained from the Medical Ethical Committee of the VU University Medical Center in 2005. Following clinical assessments at contributing mental health clinics, 687 patients aged 18 years and older with a lifetime diagnosis of OCD -regardless of the stage of the disorder, OCD subtype, presence of comorbidities, or duration of illness-, were invited to participate in the NOCDA study. Of these 687 patients, 197 (28.7%) declined to participate, 32 (4.7%) were unable to participate due to mental or physical health reasons, and 39 (5.7%) could not be contacted. The OCD diagnosis was confirmed through the Structured Clinical Interview for DSM-IV Axis I Disorders (SCID-I) (31). All respondents provided written informed consent after a comprehensive explanation of the study. Since the aim of NOCDA was to follow a large representative sample of individuals with OCD, the only exclusion criterion was an inadequate understanding of the Dutch language for the completion of interviews and self-report questionnaires. A total of 419 patients were enrolled in the study, with baseline measurements conducted between 2005 and 2009. During the follow-up period, participants received treatment as usual, which consisted of cognitive behavioral therapy and/or medication in accordance with the Dutch OCD treatment guideline (35). This guideline does not include lifestyle interventions. Assessments were conducted at two years (n=311), four years (n=295), and six years (n=272) follow-up. All research assistants underwent training and had extensive experience in assessing OCD. All interviews were audiotaped. Each research assistant’s first two interviews were monitored and a random 10% of subsequent recordings were reviewed by experienced clinicians.

### 2.2 Power of the study

The power of the NOCDA database was calculated to detect relative risks of at least 1.5. Assuming a relatively liberal loss to follow-up rate of 20% over each two-year period, the required baseline sample size was estimated at a minimum of 418 participants (see Schuurmans et al., 2021 (33)) In total, 419 participants were included at baseline, and the actual loss to follow-up remained below 20% per year.

### 2.3 Measurements

**Table 1** provides details on when specific parameters were measured.

**Table 1:** Measured parameters.

Variables	Baseline	Two-year follow-up	Four-year follow-up	Six-year follow-up	Method
Y-BOCS severity	+	+	+	+	Interview
Physical activity			+	+	Self-report
Smoking	+	+	+	+	Self-report
Using alcohol	+	+	+	+	Self-report
Using illicit drugs	+	+	+	+	Self-report
BMI	+	+	+	+	Medical examination
Comorbid depression	+	+	+	+	Interview
Medication	+	+	+	+	Self-Report

+ measurement included in the assessment.

#### 2.3.1 Physical activity

The International Physical Activity Questionnaire (IPAQ) was utilized to measure physical activity and sedentary behavior, with categorization into three levels (1 to 3) (36). An IPAQ score of 1 implies no reported activity, or some activity, but not enough to meet the second category. Category 2 implies, for example: i) five or more days per week with moderately intense activities such as bicycling at 10–12 miles per hour, recreational badminton, playing doubles tennis, or walking for at least 30 minutes a day; or ii) three days with intensive activities like playing soccer, jogging (6 miles per hour) or fast bicycling (14–16 mph) for at least 20 minutes. Individuals meeting the criteria for category 3 are most active. In accordance with Biernat et al. (37), we categorized individuals into two groups: those with i) low physical activity (IPAQ category 1); or ii) moderate or high physical activity (IPAQ categories 2 and 3). In our study, the IPAQ was measured only at the four- and six-year follow-up assessments as awareness of the relevance of physical activity to mental health increased during the study.

#### 2.3.2 Smoking, alcohol, and illicit drug use, and BMI

Questionnaires were employed to assess the following variables: smoking (yes, no), alcohol use (yes, no), weekly alcohol use, and illicit drug use (yes, no). BMI was calculated based on weight and height. Although the World Health Organization (WHO) recommends zero alcohol use, consuming small amounts of alcohol is socially accepted in many countries, and it is possible that particularly high amounts of alcohol negatively influence the course of OCD. Therefore, we analyzed both alcohol use (yes/no) and weekly consumption of standard alcohol units.

#### 2.3.3 Composite score of unhealthy lifestyle factors

To assess the cumulative impact of various lifestyle factors, we utilized a composite score of unhealthy lifestyle factors. This score was calculated based on the medical definition of healthy behaviors, which aim to reduce mortality from cardiovascular disorders and cancer, following the recommendations of the WHO (1, 2) and incorporating the avoidance of illicit drug use due to its negative effects on mental functioning (22, 25, 26). Therefore, this composite score of unhealthy factors was calculated (range 0-4) by assigning a value of 1 to smoking (yes), alcohol use (yes), illicit drug use (yes), and BMI greater than 25, while assigning a value of 0 to not smoking, not using alcohol, not using illicit drugs, and a BMI less than 25, and summing them. For the 4- and 6-year follow-ups, we added low physical activity to the total number of unhealthy factors (range 0-5), with a low IPAQ score rated as 1 and a moderate or high score rated as 0.

#### 2.3.4 Obsessive-compulsive symptoms

Severity of obsessive-compulsive symptom severity was assessed using the Yale-Brown Obsessive Compulsive Scale (Y-BOCS) (38), consisting of ten questions that evaluate the impact of time spent on obsessions or compulsions, the distress caused by them, the interference due to them, the resistance to them, and the degree of control over obsessions and compulsions. Y-BOCS scores range from 0-40 (39).

#### 2.3.5 Depressive disorder and medication

Comorbid current depression, according to the DSM-IV, was assessed through the SCID-I (34). The use of antidepressant and/or antipsychotic medication during the previous six months (yes, no) was determined using the Treatment Inventory of Costs in Patients with Psychiatric Disorders (TIC-P) (40).

### 2.4 Statistical analyses

**Figure 1:**
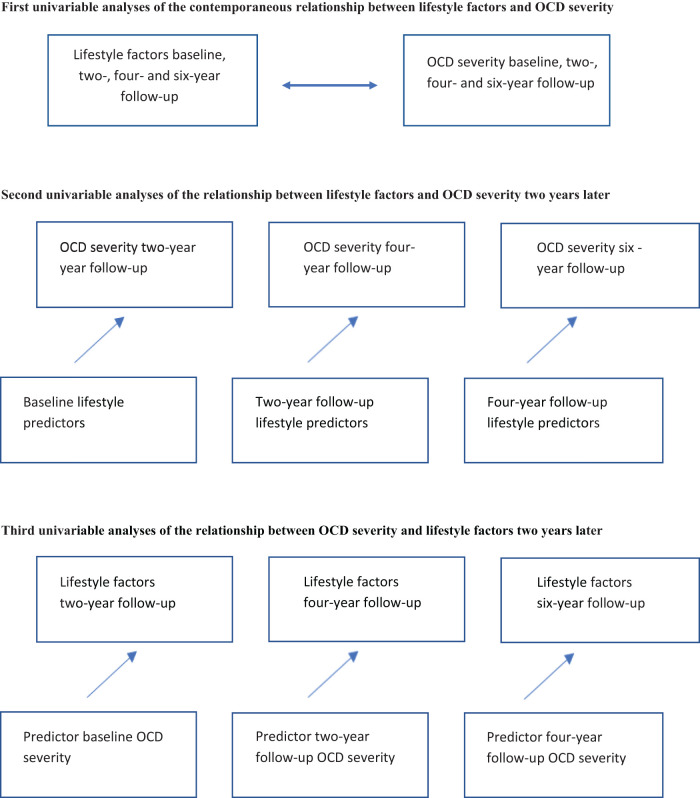
Schematic representation of the statistical analyses.

First, we conducted separate linear mixed models to describe the contemporaneous relationship between each lifestyle factor (physical activity, smoking, alcohol use, weekly alcohol consumption, illicit drug use, BMI) and OCD severity, as well as the number of unhealthy lifestyle factors and the severity of OCD. Second, we performed separate linear mixed model analyses to determine whether the presence of each lifestyle factor and the number of unhealthy lifestyle factors (independent variables) predicted OCD symptom severity (dependent variable) two years later. Third, we conducted separate linear mixed models (for continuous outcomes) and Generalized Estimating Equations (GEE) (for dichotomous outcomes) to estimate the extent to which OCD symptom severity (independent variable) predicted the presence of the measured lifestyle factors and the number of unhealthy lifestyle factors (dependent variables) two years later. Fourth, the same analyses were adjusted to account for co-occurring depressive disorders, assessing potential mediating effects. Finally, to examine potential effect modification by comorbid depressive disorders, we conducted the same analyses including an interaction between the independent variable and comorbid depressive disorder. As a sensitivity analysis, we corrected the analyses of BMI for the use of antidepressants and antipsychotic medication, as these medications are associated with higher BMI. Because the relationship between lifestyle factors and OCD severity could be influenced by sex and age, all analyses were also tested for interactions between the independent variables and both sex and age. For all analyses, *p* <.05 was considered statistically significant. Analyses were conducted using STATA (version 17). **Figure 1** provides a graphical representation of the analyses.

## 3 Results

### 3.1 Sample characteristics

**Table 2** presents the characteristics of the sample at baseline, two-, four-, and six-year follow-ups. At baseline, the total Y-BOCS score was 19.9 (8.1), indicating moderate severity of OCD symptoms. Seventeen percent of the participants were diagnosed with comorbid depression, and 69% used antidepressant or antipsychotic medication during the previous six months. Regarding lifestyle factors, at baseline, 29% were current smokers, 77% consumed alcohol, 4% used illicit drugs, and 41% had a BMI > 25. At the four-year follow-up measurement, 16.7% had a low physical activity level according to the IPAQ.

**Table 2:** Sample characteristics.

Variable	*Baseline mean (SD) or %*	*2-year measurement mean (SD) or %*	*4-year measurement mean (SD) or %*	*6-year measurement mean (SD) or %*
	N=419	N=311	N=295	N=272
Sociodemographics				
Age, years (18-79) ^a^	36.6 (10.9)			
Sex, female *Clinical characteristics*	56%			
Y-BOCS obsessions (0-20)^b^	9.9 (4.3)	7.4 (4.8)	7.5 (4.7)	7.5 (4.8)
Y-BOCS compulsions (0-20)^b^	10.0 (4.8)	7.7 (5.0)	7.9 (5.2)	8.1 (5.1)
Y-BOCS total (0-40)^b^	19.9 (8.1)	15.1 (9.0)	15.4 (9.2)	15.6 (9.4)
Comorbid depression^c^	17.2%	11.3%	11.2%	13.6%
Medication^d^	69.2%	62.3%	65.4%	63.3%
Lifestyle parameters				
Low physical activity^e^	NA	NA	16.7%	16.8%
Smoking (yes)	29%	24%	22%	21%
Using alcohol (yes)	77%	79%	79%	77%
Using illicit drugs (yes)	4%	4%	4%	4%
BMI	25.0 (5.2)	25.5 (5.4)	25.8 (5.2)	25.6 (5.3)
BMI>25	41%	45%	45%	46%
0 or 1 unhealthy factor	53%	50%	54% (45%)	54% (49%)
2 or more unhealthy factors^f^	47%	50%	46% (55%)	46% (51%)

^a^Range in dataset.

^b^Instrument range.

^c^Current depression.

^d^Antidepressant or antipsychotic medication.

^e^International Physical Activity Questionnaire (IPAQ).

^f^Unhealthy factors; current smoking-, alcohol- and illicit drug use, and BMI higher than 25. 4-year and 6-year follow-up in parentheses low physical activity is added to the unhealthy factors.

### 3.2 Results of the contemporaneous relationship between lifestyle factors and OCD severity

**Table 3a** presents the results of the univariable contemporaneous analyses of OCD severity and lifestyle factors. Smoking was significantly associated with higher OCD severity. When depression was added to the model, it partly explained the relationship between smoking and OCD severity (*β* coef = 1.3, 95% CI [-.024, 2.63], *p* = .05, Cohen’s *d*.14). Alcohol use was significantly related to lower OCD severity. Depression did not explain the relation between alcohol use, and OCD severity (*β* coef = -2.2; 95% CI -3.6 to -.88; *p* = .001, Cohen’s *d* -.25). Although there was a significant negative relation between alcohol use and OCD severity, there was no significant relationship between OCD severity and the total number of alcohol beverages per week. Other lifestyle factors were not significantly associated with OCD severity.

**Table 3a:** Contemporaneous analyses^a^ of OCD severity and lifestyle factors.

Lifestyle factor	*β Coef*	*95% CI*		*p*	*Effect size*
Physical activity^b^ (low – moderate/high)	-1.96	-4.04	.12	.07	-.22^c^
Smoking (yes)	1.65	.27	3.02	.02*	.18^c^
Using alcohol (yes)	-2.40	-3.80	-1.00	.00*	-.26^c^
Total number of alcohol beverages	-.01	-.07	.04	.65	-.02^d^
Using illicit drugs (yes)	.74	-1.85	3.33	.57	.08^c^
BMI	.04	-.08	.17	.47	.00^d^
Total number of unhealthy factors	.11	-.65	.88	.77	.01^d^
Total number of unhealthy factors inclusive physical activity^b^	-.15	-1.24	.93	.78	-.01^d^

a Performed using mixed model analyses.

b Results of the predictor analysis of lifestyle factors of the four-year follow-up measurement on Y-BOCS total score of the six-year follow-up.

c effect size Cohen’s *d*.

^d^ standardized regression coefficient.

*ρ <.05.

### 3.3 Results of the predictor analyses

**Table 3b** presents the results of the univariable analyses of potential lifestyle factors predicting OCD severity two years later, on average over a period of six years. Moderate or high physical activity predicted a significantly lower OCD severity two years later [*β* coef = -4.52, 95% CI (-7.95, -1.09); *p* <.01, Cohen’s d -.49]. The other lifestyle factors did not significantly predict OCD severity two years later. The total number of unhealthy factors did not significantly predict OCD severity after two years either. **Table 3c** shows that OCD severity did not predict any of the lifestyle factors or the composite score of unhealthy lifestyle factors two years later. Additional analyses on the influence of comorbid depression on the above-mentioned relationships showed that it did not alter these observed relationships.

**Table 3b:** Results of univariable analyses^a^ regarding lifestyle factors as potential predictors of the total Y-BOCS score two years later, on average over a time-period of six years.

Lifestyle factor	*β Coef*	*95% CI*		*p*	*Effect size*
Physical activity^b^ (low – moderate/high)	-4.52	-7.95,	-1.09	.01*	-.49^c^
Smoking (yes)	-.19	-1.82,	1.45	.82	-.02^c^
Using alcohol (yes)	-.70	-2.38,	.98	.41	-.08^c^
Total number of alcohol beverages	-.00	-.07,	.07	.96	-.00^d^
Using illicit drugs (yes)	1.66	-1.49,	4.82	.30	.18^c^
BMI	.06	-.09,	.21	.42	.04^d^
Total number of unhealthy factors	-.09	-1.02,	.84	.85	-.01^d^
Total number of unhealthy factors inclusive physical activity^b^	.60	-1.02,	2.23	.46	.05^d^

a Performed using mixed model analyses.

b Results of the predictor analysis of lifestyle factors of the four-year follow-up measurement on Y-BOCS total score of the six-year follow-up.

c effect size Cohen’s *d*.

d standardized regression coefficient.

*ρ <.05.

**Table 3c:** Results of univariable analyses regarding the total Y-BOCS score as potential predictor for lifestyle factors two years later on average over a time-period of six years.

Variables	*OR^a^/β Coef^b^*	*95% CI*		*P*	*Effect size^de^*
Potential predictor Y-BOCS severity - outcome physical activity^a^ (low – moderate/high)	1.00	.99	1.00	.36	–
Potential predictor Y-BOCS severity - outcome smoking^a^ (yes)	1.00	.99	1.01	.98	–
Potential predictor Y-BOCS severity - outcome using alcohol^a^ (yes)	1.00	.99	1.00	.42	–
Total number of alcohol beverages^b^	.00	-.00	.01	.37	.04^d^
Potential predictor Y-BOCS severity – outcome using illicit drugs^a^ (yes)	.99	.95	1.03	.57	–
Potential predictor Y-BOCS severity - outcome BMI^b^	.01	-.02	.03	.48	.01^d^
Potential predictor Y-BOCS severity - outcome total number of unhealthy factors^b^	-.00	-.01	.00	.72	-.01^d^
Potential predictor Y-BOCS severity - outcome total number of unhealthy factors inclusive physical activity^bc^	-.01	-.02	.00	.17	-.07^d^

a Performed using GEE analyses.

b Performed using mixed model analyses.

c Results of the predictor analysis of lifestyle factors of the four-year follow-up measurement on Y-BOCS total score of the six-year follow-up.

d Standardized regression coefficient.

e For the results of the GEE analyses the odds ratio’s are an effect size.

### 3.4 Additional analyses

A sensitivity analysis, in which we repeated the analyses on BMI and corrected them for the use of antidepressants and/or antipsychotic medication, did not change the results substantially. Because only a small portion of our sample used illicit drugs, we repeated the analyses on the composite score of unhealthy factors without current illicit drug use, and these analyses did not change the results either. There was no significant interaction with sex and age regarding the contemporaneous relationships between lifestyle factors and severity of OCD. Regarding the prospective relationships, on the other hand, there was a significant interaction between alcohol use and sex (*p* = .03). For female sex a negative relationship between alcohol use and OCD severity two years later was found (*β* coef = -2.3, 95% CI [-4.52, -0.09], *p* = .04, Cohen’s *d* -.25), while for male, a positive non-significant relation was found (*β* coef = 1.5% CI [-1.02, 4.06], *p* = .24, Cohen’s *d*.16). There was also a significant interaction between sex and OCD severity regarding the relationship between OCD severity and drug use two years later (*p* <.001). For female sex a negative relationship was found (OR = .91, 95% CI [.87,.95], *p* <.001), while for males a non-significant positive relation was found (OR = 1.0, 95% CI [.99, 1.08], *p* = .09). At last there also was a significant inverse interaction between age and OCD severity and drug use two years later (*p* <.01).

## 4 Discussion

The literature on lifestyle factors in OCD is limited, and to our knowledge, this is the first longitudinal study in this area. It assesses multiple lifestyle factors — including a composite score of unhealthy lifestyle factors — and examines bidirectional associations over an extended period. It aimed to examine the contemporaneous relationship between various lifestyle factors and OCD severity, to explore the association between lifestyle factors and OCD severity two years later, and to investigate the reverse relationship—the relationship between OCD severity and lifestyle factors two years later. Compared to other studies involving individuals with OCD, our sample size of 419 participants is relatively large, and the six-year follow-up period is substantial. Initially, we explored the contemporaneous connection between lifestyle factors and OCD severity. Our first hypothesis, which posited that individuals with more severe OCD would exhibit a less healthy lifestyle, was not confirmed. Our findings indicated a significant association between smoking and higher OCD symptom severity, while alcohol use was linked to a significantly lower OCD severity. However, upon adjusting for depression, the impact of smoking lost significance, while the effect of alcohol use remained independent of depression. Importantly, there was no observed correlation between the weekly number of alcoholic beverages and OCD severity, indicating the lack of a dose-response effect. In causal relationships, a dose-response relationship is often present, and the absence of such a relation suggests that the observed association may be influenced by another unknown third factor (41). Some individuals with OCD have a fear of losing control (42, 43). It is plausible that patients who fear losing control may also be hesitant to use alcohol, believing it could lead to an easier loss of control. This fear of losing control may be connected to OCD severity. In further studies on this topic, it would be interesting to consider the impact of psychological factors such as neuroticism and fear of losing control on lifestyle factors and OCD. It is also noteworthy to determine whether OCD subtypes, based on the themes of the primary obsessions and/or compulsions, are associated with different lifestyles. For example, individuals who have obsessions related to illness may have lower rates of smoking. Additionally, individuals experiencing intrusive taboo obsessions may exhibit increased fear of losing control, leading to reduced alcohol and/or illicit drug use.

Subsequently, we investigated the prospective relationship between lifestyle factors and OCD severity two years later. We anticipated that an unhealthy lifestyle would predict more severe OCD. Additionally, we expected a dose-response effect, with an unhealthier lifestyle being associated with more severe OCD symptoms two years later. This hypothesis was partly confirmed; higher levels of physical activity predicted a significantly lower OCD severity two years later. This finding aligns with a trend identified in a small randomized controlled trial (RCT) of Abrantes et al. on aerobic exercise in OCD (13), as well as with a study that found the total frequency of exercise predicted OCD symptom reduction in both a CBT with exercise and an exercise-only group (14). However, in these previous RCTs, adding exercise interventions to regular treatment did not result in significantly better treatment outcomes. Our findings suggest that increasing physical activity may only be effective for individuals with low baseline activity levels. This distinction could explain the limited impact of exercise interventions in broader OCD populations, as it is plausible that only a small percentage of these indiciuals had low physical activity levels as defined according to the IPAQ. In Abrantes et al.’s study (2017), all included participants engaged in less than 60 minutes of aerobic exercise per week at the time of inclusion. However, it is possible that these individuals had other sources of physical activity, such as walking, household cleaning, and work-related physical activity. In future studies, we recommend targeting interventions towards individuals with low physical activity levels rather than those with medium or high levels of physical activity. Additionally, directly measuring physical activity, such as through pedometers, could provide more precise data on activity patterns, potentially offering more information about within-group effects.

Although a previous study on the impact of alcohol on the course of OCD during intense residential treatment found that treatment responders exhibited lower past-year alcohol use than non-responders (15), our study did not find a positive association between alcohol use and higher OCD severity two years later. Instead, in females, alcohol use was significantly associated with lower OCD severity two years later. This prospective association, like the contemporaneous relationship between alcohol use and OCD severity, may be influenced by an unknown third factor that specific to females who consume alcohol and is associated with lower OCD severity two years later. We did not find an association between smoking, illicit drug use, and BMI and OCD severity two years later.

Finally, we analyzed the association between OCD severity and lifestyle factors two years later. We hypothesized that higher OCD severity would be associated with an unhealthier lifestyle two years later. However, we could not confirm this hypothesis. OCD severity did not predict any of these lifestyle factors and there was no association between higher OCD severity and adopting a lifestyle with more unhealthy factors. Instead, in females, a relationship was found between higher OCD severity and lower drug use two years later. The explanation for this relationship is unclear. As with the relationships found between alcohol use and OCD severity, it is possible that the prospective association between OCD severity and drug use is influenced by a third variable that may be related to both higher OCD severity and lower drug use two years later.

In various mental disorders, hypotheses have been proposed regarding the connections between smoking, illicit drug or alcohol use, and the disorders themselves. Initially, common risk factors such as specific genes and environmental factors like trauma appear to contribute to both the mental disorder and substance use and smoking (44–46). Secondly, individuals may engage in the use of illicit drugs or smoking as a form of self-medication in response to the symptoms of their mental disorders (47, 48). Thirdly, the use of illicit drugs could potentially contribute to the development of mental disorders (45, 49). Given the absence of a significantly positive contemporaneous and bidirectional prospective association between smoking, illicit drug use, alcohol use, and a higher OCD severity in our study, our findings suggest that OCD may be distinct in these aspects compared to other mental disorders. This underscores the importance of further studies exploring this relationship and the phenomenology of OCD.

The fact that current smoking, alcohol use, illicit drug use, BMI, and multiple factors of an unhealthy lifestyle were not associated with the course of OCD is clinically meaningful. This finding is encouraging for patients with an unhealthy lifestyle encompassing multiple unfavorable factors, as it suggests that an unhealthy lifestyle is not necessarily linked to worse OCD outcomes. Based on this study, healthcare providers can be just as optimistic about the expected treatment outcomes in patients with unhealthy lifestyle factors as they are for those with healthier habits.

This study has several limitations. Firstly, non-compliance - primarily because participants were unwilling or unable to participate, or not being contactable - resulted in an attrition rate of 35% over the course of six years. We compared the baseline characteristics of patients who participated in the six-year assessment and those who did not participate. Participants in the six-year follow-up did not differ from non-participants in mean Y-BOCS score, alcohol use, number of weekly alcohol consumptions, illicit drug use, BMI, use of antidepressant or antipsychotic medication, and comorbid depression. However, individuals who dropped out smoked more (χ2 (1, *N* = 410) = 14.35, *p* <.00) and used more illicit drugs (χ2 (1, *N* = 407) = 5.83, *p* = .02). There were no differences in physical activity measured at the four-year follow-up between those who dropped out between four and six years and those who participated in the six-year follow-up. Although there is a theoretical possibility that the higher dropout rate among individuals who smoked and used illicit drugs could have influenced the observed relationships between OCD severity, smoking, and illicit drug use, this concern can be mitigated. Longitudinal data analyses are specifically designed to address missing data effectively.

Secondly, we did not directly study diet but instead used BMI as a proxy. It is possible that a higher BMI may be caused by factors such as increased muscle mass. Further studies may benefit from directly measuring diet, possibly in conjunction with BMI, muscle mass, and body fat percentages.

Thirdly, we did not measure physical activity directly; instead, we relied on a self-reported questionnaire. Direct measurement of physical activity could yield different results due to over- or underreporting in self-report questionnaires. In future studies, it appears crucial to measure physical activity directly, for example by using pedometers. For smoking, using alcohol and using illicit drugs we also used self-rating questionnaires which could be biased. A study in adolescents found under-rapportage in a study on self-reported alcohol, caffeine and nicotine (50). However, another study comparing self-reports of alcohol and drug use with toxicological analyses found high agreement rates (51).

Fourthly, we followed WHO recommendations for preventing medical disorders, which advocate for abstinence from alcohol and smoking, maintaining a healthy BMI, and engaging in regular physical activity. To our knowledge, there are currently no recommendations regarding the use and quantity of alcohol, smoking, drugs, BMI, and physical activity associated with a reduced risk of psychiatric disorders.

Fifthly, lifestyle factors can be culture specific. This study was conducted in the Netherlands. More studies on this topic are needed, preferably conducted in different countries and among different cultures.

## 5 Conclusion

This study found a relationship between physical activity and OCD severity two years later. Having a low physical activity level was associated with more severe OCD. Considering that OCD, despite psychological and pharmacological treatment, is linked to high chronicity and low quality of life, it may be important to encourage individuals with OCD and low physical activity to become more active.

## Data Availability

The datasets presented in this article are not readily available because of privacy restrictions. The datasets will be made available after a data analysis plan is approved and a data sharing form is filled out. Request to access these datasets should be directed to the corresponding author(s).
